# Female Doctors

**Published:** 1857-08

**Authors:** 


*Female Doctors.*—The Virginia Medical and Surgical Journal says:—"The Massachusetts Legislature has farther endowed their Female Medical College, despite the Governor's veto. It is said that the coarseness and vulgarity of the creatures licensed by this institution is only equalled by their ignorance."

To which the New Orleans Medical News adds—after a fling at the North, which had been better omitted; for the bitterness of sectionalism has no excuse in a scientific journal.

"But, speaking of female doctors, we were struck with an observation recently made by a lady. She said that a friend of hers had been attended lately by some female doctors in New York city, and that she was congratulating herself on having escaped from their clutches. She said that during their attendance, her feelings were indescribable; she was deprived of the valuable and courteous attention of a *gentleman*, and while she felt that she was the victim of ignorance, she saw clearly that her attendant had not the slightest claim to the title of *lady*. So much vulgar talk and street gossip was never dreamed of by a genteel physician in a life-time as she heard in one short illness. And such must female doctors ever be."

Very true. The real lady is as much out of place in the practice of medicine as in a regiment of dragoons; and once begun in the rivalries of practice, with all the opportunities for scandal which a feminine taste would develop, would soon be lost in the intriguing gossip. Monthly nurses are bad enough.

